# Parenteral hydroxocobalamin dose intensification in five patients with different types of early onset intracellular cobalamin defects: Clinical and biochemical responses

**DOI:** 10.1002/jmd2.12055

**Published:** 2019-07-01

**Authors:** Emmanuel Scalais, Elise Osterheld, Christine Geron, Charlotte Pierron, Ronit Chafai, Vincent Schlesser, Patricia Borde, Luc Regal, Hilde Laeremans, Koen L. I. van Gassen, L. Bert van den Heuvel, Linda De Meirleir

**Affiliations:** ^1^ Pediatric Neurology Centre Hospitalier de Luxembourg Luxembourg; ^2^ Department of Pediatrics Centre Hospitalier de Luxembourg Luxembourg; ^3^ Laboratoire de Chimie et Hématologie Centre Hospitalier de Luxembourg Luxembourg; ^4^ Service de Biochimie, Laboratoire National de Santé Dudelange Luxembourg; ^5^ Laboratoire de Pédiatrie ULB Brussels Belgium; ^6^ Department of Genetics UMC Utrecht The Netherlands; ^7^ Department of Genetics, Radboud University Medical Center Nijmegen The Netherlands; ^8^ Pediatric Neurology and Metabolism UZ‐VUB, Vrije Universiteit Brussels Brussels Belgium

**Keywords:** early onset cobalamin metabolism defects, hemolytic‐uremic syndrome, homocysteine, methylmalonic aciduria, methylmalonic aciduria with homocystinuria, parenteral hydroxocobalamin dose intensification, subcutaneous injection port

## Abstract

Intracellular cobalamin metabolism (ICM) defects can be present as autosomal recessive or X‐linked disorders. Parenteral hydroxocobalamin (P‐OHCbl) is the mainstay of therapy, but the optimal dose has not been determined. Despite early treatment, long‐term complications may develop. We have analyzed the biochemical and clinical responses in five patients with early onset of different types of ICM defects (cblC: patients 1‐3; cblA: patient 4; cblX: patient 5) following daily P‐OHCbl dose intensification (DI). In patient 4, P‐OHCbl was started at age 10 years and in patient 5 at age 5 years. OHCbl was formulated at either, 5, 25, or 50 mg/mL. P‐OHCbl was intravenously or subcutaneously (SQ) delivered, subsequently by placement of a SQ injection port except in patient 4. In all patients, homocysteine and methylmalonic acid levels, demonstrated an excellent response to various P‐OHCbl doses. After age 36 months, patients 1‐3 had a close to normal neurological examination with lower range developmental quotient. In patient 3, moderate visual impairment was present. Patient 4, at age 10 years, had normal renal, visual and cognitive function. In cblX patient 5, epilepsy was better controlled. In conclusion, P‐OHCbl‐DI caused an excellent control of metabolites in all patients. In the three cblC patients, comparison with patients, usually harboring identical genotype and similar metabolic profile, was suggestive of a positive effect, in favor of clinical efficacy. With P‐OHCbl‐DI, CblA patient has been placed into a lower risk to develop renal and optic impairment. In cblX patient, lower P‐OHCbl doses were administrated to improve tolerability.

## 1 INTRODUCTION

Autosomal recessive disorders of intracellular cobalamin metabolism (ICM), designated as cblA‐cblJ,^1^ can give rise, either to a deficiency of methylmalonyl‐CoA mutase (cblA, cblB, and cblD variant 2) resulting in isolated methylmalonic aciduria, or to a deficiency of methionine synthase (cblD variant 1, cblE, and cblG) resulting in isolated homocystinuria or to both, (cblC, classic cblD, cblF, and cblJ), resulting in combined methylmalonic aciduria and homocystinuria. Cobalamin C (cblC) defect is the most common subgroup of inborn errors in ICM.

The spectrum of this disorder extends from fetal to adulthood period, with perinatal, early onset (EO) infantile presentation before 1 year of age, childhood (CO) (after the first year of age), adolescent and adult onset. The EO is the most frequently recognized. Infrequently (10%‐20%), at initial presentation, Cbl C patients presented a hemolytic‐uremic syndrome (HUS).^2^, ^3^ In the Caucasian population, the cblC variant c.271dupA, p.(Arg91fs), accounts for 42% of mutant alleles.^4^ In Europeans, both variants c.271dupA and c.331C>T, in the homozygous or compound heterozygous state, were primarily observed in EO cases.^3^, ^4^, ^5^ Genotype has been reported as very predictive of visual impairment, homozygous c.271dupA p.(Arg91fs) patients being severely and universally affected.^6^ P‐OHCbl is the mainstay of therapy.^2^, ^7^ In a few patients, with suboptimal metabolic control, P‐OHCbl dose escalation has been tried^8^, ^9^ in order to improve long‐term outcome. In cblA defect, caused by a pathogenic MMAA variant,^10^ chronic renal disease (CRD) may constitute long‐term sequelae, despite OHCbl therapy^11^, ^12^ and rarely, mild optic nerve atrophy.^13^ Therapy involves OHCbl.^14^ cblX defect, an X‐linked ICM disorder, with methylmalonic acidemia and homocysteinemia, is caused by a pathogenic HCFC1 variant, resulting in transcriptional dysregulation involving many genes. Despite representing a broader neurodevelopmental phenotype, severe MMACHC downregulation has been observed in cblX patients.^15^The aim of this study was to evaluate the clinical and biochemical response of P‐OHCbl‐DI in patients with different types of EO ICM defects, including cblC, cblA, and cblX.

## 2 PATIENTS AND METHODS

In five patients, with EO of different ICM defects (cblC: patients 1, 2, 3; cblA: patient 4; cblX: patient 5), clinical and biochemical responses were evaluated following daily P‐OHCbl‐DI. OHCbl was formulated at either, 5, 25, or 50 mg/mL. P‐OHCbl doses were intravenously (IV) or subcutaneously (SQ) delivered, subsequently by placement of a subcutaneous injection port (i‐Port Advance TM) to minimize cutaneous punctures except in patient 4. Neuropsychologic development was assessed by using ET 6‐6‐R^16^ in cblC patients, and WISC‐IV (verbal scale) in cblA patient. In all patients, brain MRI was performed as well as in cblC patients 1 and 3, optical coherence tomography (OCT). All procedures followed were in accordance with the Helsinki Declaration of 1975.

## 3 PATIENT 1 AND 2

**Figure 1:**
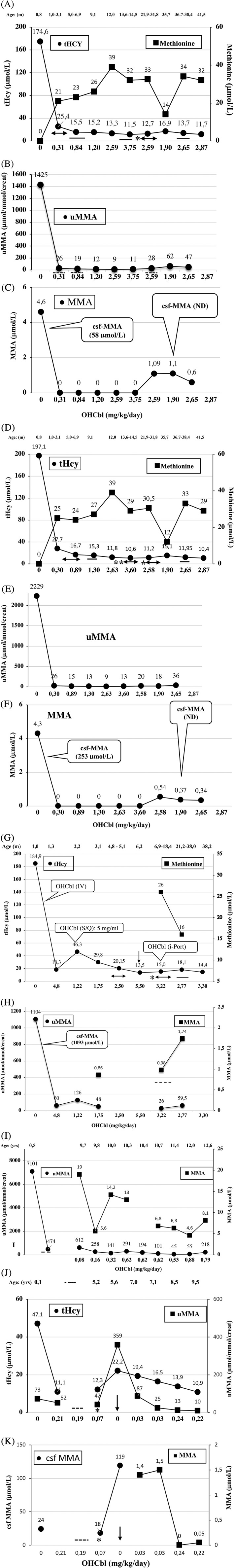
OHCbl dose reduction of tHcy, MMA, uMMA and csf‐MMA values and increase in methionine levels. Normal levels: tHcy <10.0 μmol/L, methionine 10‐40 μmol/L, uMMA <17 μmol/mmol/creat, MMA, and csf‐MMA 0 μmol/L). cblC patients 1 and 2: A and D, tHcy (black line and double arrow) levels were obtained as mean between (n = 4) age 1.0 and 3.1 months; (n = 2) age 5.0 and 6.9 months); (n = 2), 13.6 and 14.5 months; (n = 3) age 21.9 and 31.8 months); (n = 2) age 36.7 and 38.4 months). The significance has been determined by unpaired *t* test (**P = .01, *P < .05) for plasma homocysteine levels (double arrow) and is referring to the previous values (double arrow). B and E, uMMA (dotted line) levels were obtained as mean (n = 4) between age 1.0 and 3.1 months; (n = 2) between age 5.0 and 6.9 months); (n = 2) between age 21.9 and 31.8 months). C and F, MMA, initial csf‐MMA and at age 35.7 months. cblC patient 3: G, tHcy (both horizontal black line and double arrow) levels were obtained as mean between (n = 2) age 4.8 and 5.1 months; (n = 7) age 6.9 and 18.4 months; (n = 4) age 21.2 and 38.8 months). Vertical black arrow: (at age 6.2 months) double dose during 48 hours. The significance has been determined by unpaired *t* test (*P < .05) for tHcy (horizontal double arrow) and is referring to the previous values (horizontal double arrow). (H) uMMA (n = 6) and MMA (n = 5) (horizontal dotted line) levels were obtained as mean between age 6.9 and 18.4 months. Initial csf‐MMA levels. cblA patient 4: (I) uMMA (n = 7) (dotted line) levels were obtained as mean between age 6.9 and 18.4 months (range: 77‐2542). cbl X patient 5: J, dotted line: oral OHCbl *: P‐OHCbl (5 mg twice a week = 0.07 mg/kg/day) black vertical arrow: no OHCbl during 7 days. Abbreviations are as follows: m: months; yrs: years; tHcy: plasma total homocysteine; (u) (csf) MMA: (urinary) (cerebro‐spinal fluid) methylmalonic acid; ND: not detected; IV‐OHCBL: intravenous hydroxocobalamin, S/Q‐OHCBL: subcutaneous hydroxocobalamin (formulated at 5 mg/mL and 25 mg/mL); i‐port: subcutaneous injection port (i‐port advance TM) (formulated at 25 mg/mL and/or 50 mg/mL). OHCbl blood levels: (dose of 2.41 mg/kg/day: 17.5 × 10^6^ pg/mL in cblC patient 1 and 17.0 × 10^6^ pg/mL in cblC patient 2); (dose of 2.98 mg/kg/day: 11.0 × 10^6^ pg/mL in cblC patient 3); (dose of 0.88 mg/kg/day: 4.80 × 10^6^ pg/mL in cblA patient 4); (dose of 0.21 mg/kg: 3.40 × 10^6^ pg/mL in cblX patient 5) (normal <1999 pg/mL)

Since birth, patients 1 and 2, twin girls, presented anorexia, lethargy, failure to thrive, neutropenia (240 and 370 G/L, normal >1500) and nystagmus. On admission, at age 3 weeks, both plasma total homocysteine (tHcy) and urinary methylmalonic acid (uMMA) values were elevated. Subsequently, both infants were treated (day one) with P‐OHCbl (0.31 mg/kg/day) supplemented with oral betaine (100 mg/kg/day), folinic acid (5 mg/day), and oral L‐carnitine (50 mg/kg/day), which was followed by clinical improvement. The biomarker response, at onset and following P‐OHCbl‐DI, is represented in Figure 1A‐F. Molecular analysis revealed homozygosity for the c.271dupA (p.Arg91fs) variant. Twin zygosity DNA testing confirmed monozygotic twins. At age 32 months, in both patients, no abnormal eye movements, such as nystagmus, were observed, except in patient 1, mild intermittent, small ocular jerks. Daily P‐OHCbl dose was 2.40 mg/kg. In both siblings, the neurological examination was normal except for hypotonia. Patient 1 walked independently at age 20 months and patient 2 at age 25 months. At age 3 years, both infants could pronounce three‐word sentences in two languages and use two personal pronouns. Both patients could build a tower of more than six blocks, master her pincer grasp with right‐hand preference and making circular and both horizontal and vertical line with three‐digital grasp in patient 1 and palmar grasp in patient 2. Results of developmental, ophthalmologic and MRI evaluations are shown in Table 1.

**Table 1:** Characteristics of reported EO cblC patients and the three EO cblC studied patients with usually identical genotype and similar metabolic profile

									Neuropsychological test: aDQ, bVABS, cWAIS‐III, dET 6‐6‐R						
Age of onset (wk)	tHcy	Neur	Visual function (at most recent exam)				MRI (age at study time)	c.DNA	GM	FM/DLS	Communication	Cognitive	Social	Behav	References
			N	ONP/A	PR	Acuity									
								NR	a37 ± 17***	53 ± 29		45 ± 18			Anderson et al^17^
	40*	EP, hy, μ	+		+	↓	Thinned CC‐WM‐GM‐↑T2‐WM, VM,↑T2‐BG (27 mo)	NR	b< p1	b< p1	b< p1	b< p1	b< p1	b< p1	Enns et al^18^
2	186			+/+	−	↓	Thinned WM, moVM	c.271dupA/c.271dupA	↓	↓	↓	↓	↓	Autistic	Sharma et al^19^
NBS		hy	+	+/−	+	FAF	Thinned CC‐WM (pv) (81 mo)	c.271dupA/c.271dupA	b25	20	33		48	31	Weisfeld‐Adams et al^20^
NBS		hy	+	+/+	+	CSM	↑T2WM (pa) (49 mo)	c.271dupA/c.271dupA	97	105	72		97	91	
NBS		hy, μ	+	NR	NR	NR	NP	c.271dupA/c.271dupA	72	85	85		79	77	
NBS		hy	+	**	−	FAF	NP	c.271dupA/c.271dupA	90	75	75		82	78	
NR	NR	E	+	+/+	+	Fx	miA (cerebrum, cerebellum)	c.271dupA/c.271dupA			cV:61	P:49			Bellerose et al^21^
4	203	hy	+	−	+	Fx	Thinned CC‐WM	c.271dupA/c.271dupA	bp3	p2	p3		p4	p1	
6	NR	μ, E	+	+/+	+	LP	Thinned CC‐WM	c.271dupA/c.271dupA	b< p1	< p1	< p1		< p1	< p1	
3	40‐110	E, hy	+	BEM	+	CuSuM	ND	c.271dupA/c.271dupA	↓(severe)		↓ (severe)			↓	Ku et al^13^
1^$^	60‐100	DD	+	BEM	−	CSM	ND	c.271dupA/c.271dupA			↓(mild)				
3	174.6	hy	−	−	−	CSM^^^	↑T2WM (3 wk), Nl^+^(36 mo)	c.271dupA/c.271dupA	d63	83	83	66‐83	83	Nl	This study
3	197.1	hy	−	−	−	CSM^^^	↑T2WM (3 wk), Nl^+^(36 mo)	c.271dupA/c.271dupA	63	83	83	66‐83	83	Nl	
4	184.9	−	+	−	−	CuSM^++^	↑T2WM, VM (2 mo) thinned CC‐WM, ↓VM (18 mo), My	c.271dupA/c.271dupA	87‐100	87‐100	87	87	87	Nl	

*Note*. *: homocystine levels (normal 0 μmol/L); **: macular lesion; *** mean ± SD; +: except mild ↑T2 periventricular parietal region; ++: electroretinogram: ↓ scotopic and photopic response and optical coherence tomography (OCT): thin intra‐retinal cysts and irregularity of photoreceptors (both performed at age 27 months and stable at age 36 months); ↑T2: increase signal intensity in T2 sequence; ^: OCT performed at age 3^2/12^ years: mild reduction of the external nuclear layer in the parafoveolar region; $: mother treated with P‐OHCbl at her third trimester.

Abbreviations: BG, basal ganglia; CC, corpus callosum; CSM, central/steady/maintained vision; CuSM, central, unsteady, maintained vision; CuSuM, central, unsteady, unmaintained vision E: epilepsy; EP, extra‐pyramidal signs (progressive choreo‐athetosis); FAF, fixes and follows; Fx, fixates; GM, gray matter; Hcy, homocysteine levels (normal <10.0 μmol/L); hy, hypotonia; LP, light perception; miA, mild atrophy; moVM, moderate ventriculomegaly; My, gain in myelination; mo, months; μ, microcephaly; Neur, neurological status; N, nystagmus; NBS, newborn screening; Nl, normal; NP, not performed; ONP/A, optic nerve pallor/atrophy; PR, pigmentary retinitis; (pv) (pa), (periventricular) (periatrial); < p, less than percentile; WM: white matter; wk, week.

a DQ: developmental quotient: GM: gross motor; FM: fine motor.

b VABS: Vineland Adaptive Behavior Scale; DLS: daily live scale; Ction: communication; Social: socialization; Behav: adaptive behavior index.

c WAIS‐III: Weschler Adult Intelligence Scale‐III: V: verbal IQ; P: performance IQ.

d ET 6‐6‐R: assessment of a developmental status for children from 6 months to 6 years‐revision.

## 4 PATIENT 3

At age 3 weeks, cblC patient 3, a boy, presented anorexia and peri‐orificial dermatitis. He was admitted at age 27 days with multi‐organ failure and metabolic acidosis (anion gap: 21 mmol/L, normal 8‐16). He had a HUS, characterized by the triad^22^ of hemolytic anemia (8.0 g/dL, normal>10), uremia (80‐117 mg/dL, normal <50) and thrombocytopenia (10 G/L, normal >230). On blood smear, schizocytes (2%) as well as neutropenia (620 G/L, normal >1500) were found. Following a high tHcy level, (at day 2), IV OHCbl (4.1 mg/kg/day) was administrated, supplemented with oral betaine (150‐200 mg/kg/24 hours) and both IV folinic acid (5 mg) and L‐carnitine (100 mg/kg). In spite of infrequently used,^23^ oral methionine dose was initially started at 33 mg/kg/day and interrupted at age 10 months. The biomarker response at onset and following P‐OHCbl‐DI is reported in Figure 1G,H. Molecular analysis revealed the homozygous c.271dupA (p.Arg91fs) variant. Progressively, multi‐organ failure resolved. At age 5 months, he had cholangitis (total biliary acid levels 20.5 μmol/L, normal <10). P‐OHCbl doses were increased from a mean value of 2.50 to 3.20 mg/kg/day resulting in normal total plasma biliary acid levels at age 22 months (3.3 μmol/L, normal<10). Despite moderate visual impairment with right head tilt and nystagmus, visual performance improved both in the ability to fix and follow. He walked independently at age 20 months. At age 24 months, he could speak several words in two languages and use onomatopoeia and gestural support (bye, bravo, pointer finger such as designate familiar objects). Results of developmental, ophthalmologic, and MRI evaluations are shown in Table 1.

## 5 PATIENT 4

Patient 4, a boy, was born at term from non‐consanguineous parents. He started to walk at age 18 months and speak sentences at 2½ years. Before admission, he was diagnosed as hypotonic with poor head control. At age 6 months, he showed metabolic acidosis with hyperketonemia. uMMA levels were markedly increased. Molecular analysis revealed a compound heterozygous variant in MMAA gene located on chromosome region 4q31.21, c.439+4_439+7del, p.? inherited from the mother and c.593_596del, (p.Thr198fs) inherited from the father. Initially, he was treated with dietary protein restriction supplemented with oral OHCbl (1 mg). He had several episodes of metabolic acidosis during intercurrent febrile infections. At age 9 years, daily P‐OHCbl was initiated. The biomarker response, at onset, at age 9 years, and following P‐OHCbl‐DI is reported in Figure 1I. At age 10 years, his physical and neurological examination was normal. Ophthalmological examination was normal including eye fundus and visual field as well as his renal function. Performed at age 11½ years, both brain MRI and MR spectroscopy were normal. Cognitive level was also normal at age 8 years (WISC‐IV: verbal scale 99).

## 6 PATIENT 5

Patient 5, a boy, previously reported,^24^ showed, at age 10 days, refractory seizures and high CSF glycine mimicking non‐ketotic hyperglycinemia. Both urinary methylmalonic acid and plasma homocysteine were elevated. At age 5 years, he was diagnosed to be affected by the cblX defect. He resulted to carry a variant, in the global transcriptional coregulator, *HCFC1*. A hemizygous variant, c.2728A>G, (p.Lys910Glu) was also detected in *ATRX*. Clinically, this patient was characterized by severe developmental disability and intractable epilepsy. P‐OHCbl (0.53 mg/kg/week) produced seizure and or involuntary movement exacerbation, despite metabolic improvement. However, macrocytosis was not corrected (mean corpuscular volume [MCV] 103.2 μ^3^, normal 76‐91). At age 7 years, following a very slow increase in P‐OHCbl doses, as shown in Figure 1J,K, a better improvement in seizure control was obtained, but not in neurological development. Macrocytosis, although less marked, was still present (MCV 99.5 μ^3^ vs 105.4 and 106.6, normal 76‐91) suggesting that his P‐OHCbl dose was not yet sufficient.

## 7 DISCUSSION

The three cblC patients harbor the most frequent variant which is, in the Caucasian population, c.271dupA (p.Arg91fs), located in the MMACHC gene. Being homozygous, they belong to the EO with the highest risk to develop maculopathy, progressive retinopathy and cognitive delay.^6^, ^25^, ^26^ Usually, well treated infants, maintained, on follow‐up, moderately elevated plasma tHcy levels (20‐80 μmol/L) as well uMMA (10‐100 μmol/mmol/Cr).^1^ In contrast, in the three cblC patients, P‐OHCbl‐DI produced excellent biochemical responses such as lower range plasma tHcy and higher methionine values (Figure 1A,D,G) as well as low uMMA, csf‐MMA and MMA levels (Figure 1B,C,E,F,H). Compared to cblC patients 1 and 2, cblC patient 3, initially required higher OHCbl doses, to control plasma tHcy values. This discrepancy could be related to disease severity, including HUS, multi‐organ failure, metabolic acidosis and poor perfusion. Not surprisingly, before hospital discharge, at age 2.2 months, P‐OHCbl, being formulated at 5 mg/mL, had to be temporally administrated at lower dose (1.22 mg/kg), which raised plasma tHcy and urinary MMA levels (Figure 1G,H). Subsequently, by using P‐OHCBL‐DI (2.50 mg/kg), formulated at 25 mg/mL and 50 mg/mL, plasma tHcy and uMMA levels decreased to lower values. At age 6.2 months, cblC patient 3 received, following medical order misinterpreted, double P‐OHCbl dose (5.50 mg/kg/day), for 48 hours. Subsequently, plasma tHcy dropped from 22.5 to 13.5 μmol/L (Figure 1G). However, how can we assume that such as P‐OHCbl‐DI could have had therapeutic effects on the three cblC patients? Although, the three cblC patients were promptly treated with P‐OHCbl‐DI, they were initially already symptomatic and EO CblC defect presents both developmental and degenerative features. Therefore, it was not surprising to have observed some degree of developmental and ocular manifestations in the three cblC patients. Our study was limited by the small number of patients who could not be randomized to different treatment doses. However, we found indirect evidence for the efficacy of higher doses of OHCbl. Following P‐OHCbl‐DI, nystagmus disappeared in cblC patients 1 and 2 and visual behavior significantly improved in cblC patient 3 with no sign of ocular disease progression at age 3 years. Compared to previous studies (Table 1), most of the EO cblC patients, usually harboring a homozygous c.271dupA (p.Arg91fs), with high initial tHcy (>60 μmol/L) or high homocystine (>30 μmol/L) levels and treated at onset with low P‐OHCbl dose, have prominent developmental delay, autistic features, pigmentary retinitis, optic atrophy, marked MRI abnormalities (ventriculomegaly, WM atrophy) and persistent moderately elevated plasma tHcy levels.^17^, ^18^, ^19^, ^20^, ^21^ Importantly, homozygous c.271dupA patients with, at onset, low plasma tHcy (<60 μmol/L) or homocystine (< 30 μmol/L), may have a better prognosis.^27^

Similarly, the response of HUS to higher‐doses of OHCbl in two CO cblC siblings has been specified.^28^ These siblings required a serum cobalamin concentration of approximately 1 × 10^6^ pg/mL to obtain clinical and biochemical responses. In another study,^8^ an EO cblC patient, with PR and mild developmental delay received, at 1‐month interval, an increasing dose regimen (0.35 mg/kg). This strategy resulted in an 80% and 55% reduction of plasma MMA and tHcy, respectively. Five other EO cblC patients,^9^ aged between 5 and 14 years, received also high P‐OHCbl doses (0.1 mg/kg/day to 0.35 mg/kg/day), leading in patient 1 and patient 2 to reduction of tHcy (30% and 17%).

In the cblA patient, the c.439+4_439+7del p (?) variant inherited from the mother was predicted to delete the canonical splice site of exon 2.^29^ Being at risk, before P‐OHCbl‐DI, to develop later mild optic neuropathy and/or CRD, because of previous uMMA levels above the approximate threshold of 2000 μmol/mmol/creatinine,^12^, ^13^ the cblA patient 4 had normal visual and renal function on early follow up. The cblX patient responded biochemically and clinically only after slowly increasing the P‐OHCbl dose and eventually achieved better seizure control. The poor clinical outcome could be related mainly to the HCFC1 variant, being a transcription factor involving many genes, and to a lesser extend to the ATRX variant.

In conclusion, this study showed an excellent biochemical response in all patients, and without significant side effects. Among the metabolic biomarkers, plasma tHcy was the most sensitive one. OHCbl dose‐response relationship was only observed with plasma tHcy levels. Sustained absolute normal biomarker values were not obtained. In comparison with EO cblC patients usually harboring identical genotype and similar metabolic profile, we observed a better than expected outcome in the three cblC patients, suggestive of a positive effect of P‐OHCbl‐DI in favor of clinical efficacy, in particular, in view of the absence of progressive retinopathy, a more favorable cognitive outcome and improved brain myelination. With P‐OHCbl‐DI, CblA patient has been placed into a lower risk to develop CRD and or mild optic atrophy. In cblX patient, very slowly progressive increase in P‐OHCbl dose was necessary to improve clinical tolerability and control seizures. Nevertheless, long‐term follow‐up of both cblC and cblA patients as well as larger population are needed to confirm this potential efficacy of P‐OHCbl‐DI. High‐dose of P‐OHCbl was also easier to administer to the child when P‐OHCbl was formulated at 50 mg/mL and delivered by a SQ injection port.

## CONFLICT OF INTEREST

None declared.

## AUTHOR CONTRIBUTIONS

E.S. wrote the article, supervised the findings of this work, devised the project, and the main conceptual ideas. L.D.M. and L.R. supervised the metabolic evolution and worked on the article and revised the article. C.G. and C.P. evaluated the children initially and revised the article. R.C. and E.O. revised the article. H.L., P.B., and V.S. performed the biochemical analysis. K.L.I.v.G. and L.P.W.J.v.d.H. performed molecular analysis. All authors discussed the results and contributed to the final article.
